# Cost-Effectiveness Comparison of Genechip and Conventional Drug Susceptibility Test for Detecting Multidrug-Resistant Tuberculosis in China

**DOI:** 10.1371/journal.pone.0069267

**Published:** 2013-07-23

**Authors:** Yu Pang, Qiang Li, Xichao Ou, Hojoon Sohn, Zhiying Zhang, Junchen Li, Hui Xia, Kai Man Kam, Richard J. O’Brien, Junying Chi, Shitong Huan, Daniel P. Chin, Yan-lin Zhao

**Affiliations:** 1 National Center for Tuberculosis Control and Prevention, Chinese Center for Disease Control and Prevention, Beijing, China; 2 Independent consultant, Daegu, Republic of Korea; 3 PATH, Beijing, China; 4 Stanley Ho Centre for Emerging Infectious Diseases, Faculty of Medicine, Chinese University of Hong Kong, Shatin, Hong Kong, China; 5 Foundation for Innovative New Diagnostics (FIND), Geneva, Switzerland; 6 Bill and Melinda Gates Foundation, China Office, Beijing, China; St. Petersburg Pasteur Institute, Russian Federation

## Abstract

**Background:**

Genechip (CapitalBio, Beijing, China) is a system for diagnosing resistance to rifampin and isoniazid, which shows high efficiency in detecting drug-resistant tuberculosis. Here, we firstly evaluated the costs of Genechip for detecting the drug susceptibility of *Mycobacterium tuberculosis*, compared to conventional drug susceptibility test (DST) in laboratories in China.

**Methodology/Principal Findings:**

Data on the costs of the two tests were collected at four hospitals. Costs were calculated using the essential factor cost calculation method. The costs of diagnosing a single case of multidrug-resistant tuberculosis (MDR-TB) using Genechip and DST were US$22.38 and $53.03, respectively. Taking into account the effect on costs from failure of a certain number of tests to accurately diagnose MDR-TB, the costs of Genechip and DST increased by 17.65% and 5.22%, respectively. The cost of both tests decreased with the increasing prevalence of MDR-TB disease, and the cost of Genechip at a sensitivity of more than 50% was lower than that of DST. When price of Genechip was varied to 50%, 80%, 150%, and 200% of the original price, the cost of Genechip at sensitivities of more than 30%, 40%, 60%, and 70%, respectively, was also lower than that of DST.

**Conclusions/Significance:**

This study showed that Genechip was a more cost-effective method of diagnosing MDR-TB compared to conventional DST.

## Introduction

Multidrug-resistant tuberculosis (MDR-TB) is a serious challenge to efforts to control tuberculosis (TB) all over the world [1]–[3]. The World Health Organization estimates that about 440,000 new cases of MDR-TB are diagnosed every year, with about 50% of those in India and China [2], [3]. Early diagnosis is essential in controlling transmission of MDR-TB and provides reference results for developing effective treatment regimens [4], [5].

Conventional drug susceptibility testing (DST) is the gold standard for determining anti-TB drug susceptibility of *Mycobacterium tuberculosis* (MTB) [6]–[8]. However, because MTB grows slowly, it takes about two to three months to complete the test [9]–[10]. It is possible to shorten the time required for diagnostic results by using a liquid media culture system, but this method still requires at least 10 days [7]. Drug resistance may also be diagnosed by scanning the genes associated with drug resistance, using molecular tools that shorten the time required for diagnosis [11]–[13].There are several commercial kits available for diagnosing drug resistance of MTB using molecular tools, including MTBDRplus (Hain Life science GmbH, Germany), INNO-LiPA RifTB (Innogenetics, Belgium), and GeneXpert (Cepheid, USA) [11], [14]–[16].

Similar to three other commercially available kits, Genechip is a system for diagnosing resistance to rifampin and isoniazid, the two most effective drugs against MTB, by detection of frequent mutation codons in *rpoB*, *katG*, and *inhA* promoter regions [17]. This molecular diagnostic tool shows high efficiency in detecting drug-resistant MTB, with sensitivity and specificity to rifampin resistance of 94.3% and 94.7%, respectively [17], [18]. Previous studies have shown that the excessive diagnostic cost associated with the molecular biological method may serve as limitation to implementation on a wider scale [19]–[21]. However, limited published data are available on cost associated with both conventional DST and Genechip for detecting anti-TB drug resistance.

The objective of this study is to analyze the costs of Genechip compared to conventional DST in a range of hospital laboratory settings in China. We also investigated the influencing factors, which may impact the unit cost of Genechip.

## Materials and Methods

### Ethics

The study was supported by the Bill & Melinda Gates Foundation. The study design was approved by PATH and the ethics committee of the Foundation for Innovative New Diagnostics. All patients signed a consent form before being included in this study. The National Reference Laboratory of Tuberculosis of the Chinese Center for Disease Control and Prevention is responsible for this project.

### Design of Study

This study was performed in four hospitals belonging to four prefectural cities in China, including the Fourth Hospital of Inner Mongolia, Hohhot, Inner Mongolia Autonomous Region; Tuberculosis Dispensary of Kaifeng, Henan Province; the Fourth Hospital of Lianyungang, Jiangsu Province; and Yongchuan Hospital, affiliated to Chongqing Medical University, Yongchuan District, Chongqing Municipality. The Fourth Hospital of Inner Mongolia Autonomous Region and the Fourth Hospital of Lianyungang City, Jiangsu Province, are specialized hospitals for infectious diseases, while the Tuberculosis Dispensary of Kaifeng City, Henan Province, is a small, specialized tuberculosis hospital. Yongchuan Hospital in Chongqing is a high-level, comprehensive hospital. These pilot sites represented the northern, eastern, central, and western regions of China, respectively. The cities belong to different administrative divisions in China and represent varying levels of economic development.

Cost analyses on Genechip and conventional DST were performed separately in four local laboratories. All laboratories in the four sites were retrofitted ahead of carrying out the project to meet the national standards for clinical molecular testing. The costs of modifying the laboratories for the conventional DST and Genechip were about US$20,000 and $75,000, respectively, while those for purchasing instruments were about $10,000 and $36,000, respectively.

### Laboratory Method

The Genechip was performed according to the instructions provided by the manufacturer [17]. The procedure for performing conventional DST was as follows: The sputum specimen was digested with 4% sodium hydroxide at an equal volume for 15 minutes and inoculate to L-J solid media. After six to eight weeks of culture, the bacterial colonies were collected for conventional DST as previously reported [22].

### Cost Analysis

The costs of Genechip and conventional DST were estimated by the time-observation method according to the literature reported previously [20], [23]. All costs were collected from the time a specimen digested at the laboratory until the time test results were available. The costs include those for the laboratory space, building, equipment, staff salary, reagent, and consumables as well as quality control and equipment maintenance. The cost data were obtained by various methods, including investigation, interview, and field collection. In addition, specimen sizes in small, medium, and large quantities were analyzed separately in the four laboratories to evaluate costs based on sample size. The mean of the costs in the four project sites served as the final cost.

The cost of diagnosing a single case of MDR-TB was calculated according to disease prevalence rates in various regions. The cost of Genechip for diagnosing a single case of MDR-TB was calculated based on a purchase price for the diagnostic kit of US$11.03.

## Results

### Average Unit Cost of Conventional DST

Our analysis found that the mean cost of diagnosing a single case of MDR-TB by conventional DST was $53.03 (Table 1). The costs of reagent, consumables, and overhead formed the largest share of the total cost, while the costs for the building and instruments formed a smaller share. Across the four project sites, we found that the costs in Hohhot, Lianyungang, and Yongchuan were similar, while costs in Kaifeng were lower. Additional analysis showed that all costs in Kaifeng were lower, with the costs of management and consumables lower, than the costs in other regions. Costs varied in the other three regions, with the cost of consumables highest in Hohhot and the cost of personnel highest in Yongchuan.

**Table 1:** Average unit cost of conventional DST.

Input type	Cost per unit (2011 US$)				
	Hohhot	Lianyungang	Kaifeng	Yongchuan	Mean (%)
Overhead	10.89	13.27	5.68	11.55	10.35(19.51)
Building	4.04	3.56	3.21	3.09	3.47(6.54)
Equipment	1.21	1.33	1.05	1.68	1.32(2.49)
Staff	3.31	4.36	2.1	11.7	5.37(10.12)
Reagents and chemicals	20.09	20.3	15.93	20.65	19.24(36.28)
Consumables	18.04	12.19	10.49	12.39	13.28(25.04)
Total	57.57	55.02	38.46	61.06	53.03(100)

### Average Unit Cost of Genechip

The mean cost of diagnosing a single case of MDR-TB using the Genechip test was $22.38 (Table 2). The costs of reagent, consumables, and management made up more than 90% of the total cost, with the cost of reagents alone accounting for 60.14% of total cost. The costs across the four project sites were similar, with highest overall costs in Liangyungang and lowest costs in Kaifeng. The costs of personnel and consumables in Kaifeng were lower than those in the other three cities.

**Table 2:** Average unit cost of Genechip.

Input type	Costs per test (2011 US$)				
	Hohhot	Lianyungang	Kaifeng	Yongchuan	Mean(%)
Overhead	3.30	4.16	2.46	1.95	2.97(13.27)
Building	0.02	0.03	0.03	0.02	0.03(0.13)
Equipment	0.33	0.33	0.28	0.51	0.36(1.61)
Staff	1.19	1.72	0.96	2.55	1.6(7.15)
Reagents and chemicals	13.23	13.77	13.26	13.57	13.46(60.14)
Consumables	4.03	4.35	3.24	4.21	3.96(17.69)
Total	22.11	24.37	20.23	22.81	22.38(100)

### Cost after Taking Actual Loss into Account

We performed a separate analysis to take into account the effect on cost from the failure of a certain number of tests to accurately diagnose MDR-TB and the resulting impact on the validity rates of both tests. In some cases, specimens are positive in the smear examination but negative in the actual clinical test. When the failure rate (false negative) of the test was 91.48%, and the validity rate of conventional DST was 99.0% (the true average value of four pilots), the cost of conventional DST was calculated as $55.80, a 5.22% increase compared to the cost before taking the loss into account (Table 3). When the validity rate of a single Genechip was 85%, the cost of the Genechip was $26.33, an increase of 17.65% compared to the cost before taking the loss into account. However, during the actual application of Genechip, the loss usually has more powerful effect on the cost.

**Table 3:** Cost of diagnosing a single case of MDR-TB, taking loss into account.

Input type	Costs per test (2011 US$)	
	DST^a^	Genechip^b^
Overhead	11.00	3.49
Building	3.70	0.03
Equipment	1.38	0.43
Staff	5.73	1.89
Reagents and chemicals	19.94	15.83
Consumables	14.05	4.66
Total	55.80	26.33

a The cost of DST calculated based on a rate of specimens positive in smear examination and negative in culture of 91.48% and a validity rate of 99%;

b The cost of Genechip calculated based on a validity rate of 85%.

### Cost of Diagnosing a Single Case of MDR-TB

**Figure 1:**
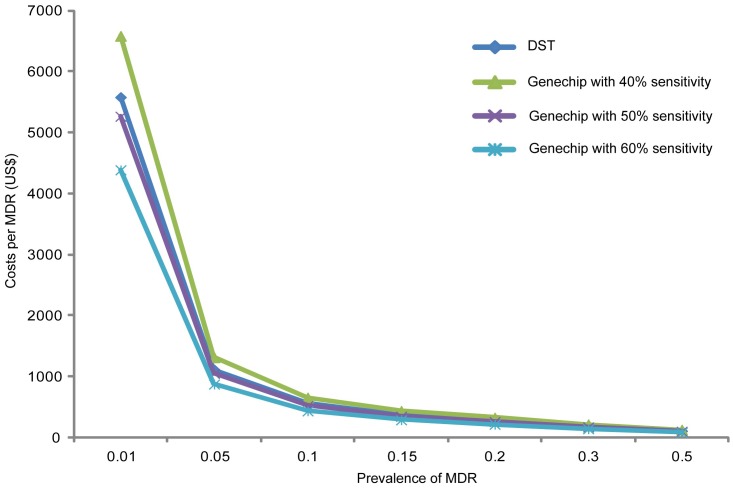
Effect of sensitivity and MDR-TB prevalence on cost of diagnosis using Genechip and conventional DST.

The prevalence of MDR-TB directly influences the cost of diagnosing the disease [24]. Similarly, the sensitivity of the diagnostic method also influences cost. We analyzed the costs of conventional DST and Genechip for diagnosing a single case of MDR-TB, using conventional DST result as a gold standard. As shown in Figure 1, the costs for both Genechip and conventional DST decreased with increasing prevalence of MDR-TB, while Genechip cost also decreased with increasing sensitivity of the method. Genechip cost was higher than conventional DST when sensitivity was 40%, but when sensitivity was not less than 50%, Genechip’s cost was lower than conventional DST.

### Effect of Genechip Price and Sensitivity on Diagnosis of a Single Case of MDR-TB

**Figure 2:**
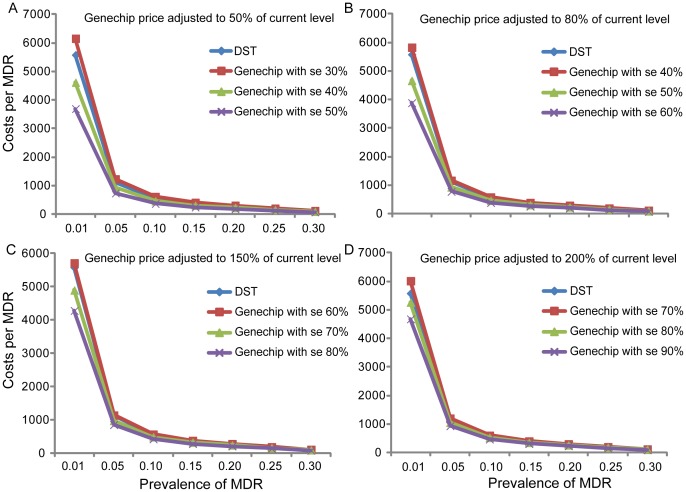
Effect of the price of Genechip on the cost of diagnosis. A–D represents the costs of diagnosing a single case of MDR-TB by Genechip with various sensitivities at various prevalence rates when the price of Genechip is adjusted to 50%, 80%, 150%, and 200% of the current price level.

Cost analysis showed that the main cost of Genechip was that of reagents ($11.03 per unit). To assess how product price influences the cost of diagnosis, we analyzed the effect of adjusting the price of Genechip. As shown in Figure 2A, when the price of Genechip was 50% of the original price, the cost of diagnosing MDR-TB using conventional DST was lower than the cost for using Genechip, except when the sensitivity was more than 40%. Similarly, when the price of Genechip was adjusted to 80%, 150%, and 200% of the original price, the cutoff values of sensitivity of Genechip by which the cost of diagnosis was lower than that by conventional DST were 50%, 70% and 80%, respectively. With the expected increase in price of Genechip, the sensitivity should be improved in order that the average cost of detecting one MDR case would be lower than conventional DST.

## Discussion

Various factors influence the cost of the two diagnostic methods. First, local economic conditions directly influence the cost. We found that all costs in Kaifeng were lower than those in the other three project sites, mainly due to the relatively low level of economic development in Kaifeng and the resulting lower costs of personnel and management. We observed that as the turn around time of the diagnostic method increases, the cost difference also increased. This effect was in addition to variations in local economic conditions.

Second, the class of hospital where the test was performed influenced the diagnostic cost. In China, the salary of a doctor was associated not only with local economic conditions but also with the grade of hospital in which he/she was employed. In general, salaries in hospitals were at high levels or in comprehensive hospitals are higher than those at lower levels or in specialized hospitals. The project site in Kaifeng was a small hospital specializing in TB, resulting in the lower salary costs than those in the other three sites. The project site in Yongchuan was a comprehensive, class AAA hospital, resulting in higher salary costs than those in other sites. The difference was even greater with conventional DST.

Finally, the difference in the purchase price of consumables in the four project sites also influenced the cost of diagnosis. In this study, the Genechip kits and culture and DST media used in various sites were purchased uniformly, while the consumables were purchased individually, resulting in a difference in purchasing price that was directly reflected in the cost of diagnosis. For example, the price of consumables for biosafety in Hohhot was the highest in the four cities, resulting in a higher cost of DST diagnosis than in other sites.

Inevitably, a certain proportion of results failed to be diagnosed (“false negatives”) during application of the diagnostic kit. Failures in culture for conventional DST at both the early and late stage of diagnosis may increase the cost of this method. Similarly, a certain proportion of results showed failure of diagnosis was found during application of Genechip, and these failures must be taken into account in calculating the unit cost of diagnosis. In this study, after taking the losses into account, the increase in the cost of Genechip was higher than that of conventional DST (17.65% vs. 5.2%), indicating that Genechip diagnostic protocol should be further optimized by manufacturer to decrease product cost. However, even taking losses into account, we found that the overall cost of diagnosing a single case of MDR-TB using Genechip was lower than the cost of conventional DST.

The local prevalence of MDR-TB must be taken into account in calculating diagnostic cost, so that the cost of diagnosing all patients, whether they were tested positive or negative for MDR-TB, was included in the total cost. As reported previously [17], the sensitivity of Genechip for diagnosing MDR-TB was about 75%, indicating that if the market price of Genechip is not more than $22.06, the cost of Genechip is lower than that of conventional DST.

In addition to the lower cost, Genechip has other advantages as compared to conventional DST [17]. Firstly, the time for diagnosis using Genechip is about six to eight hours, considerably shorter than the two to three months required when using conventional DST. Second, as compared with conventional DST, Genechip provided better biosafety. As reported elsewhere, the risk of infection with TB in laboratory personnel using conventional DST is more than 20 times higher than that for personnel using smear examination [25]. Genechip is a nucleic acid-based method with superior safety compared to conventional DST. Taking these factors into account as well as cost, Genechip may be a more cost-effective method.

There are several limitations to this study. Firstly, sputum smear was not an ideal gold standard, and false negative rates using this definition would be lower than the true false negative rate, as smear- negative TB cases would also be missed by both assays. Secondly, although the costs of the two methods were analyzed, the benefits of each method were not analyzed in this study. Thirdly, the cost of diagnosing a single case was only analyzed based on the cost of the diagnostic method used. However, a method with a sensitivity of less than 50% has no practical significance in field application. Moreover, the limitation of Genechip included the loss of knowledge regarding resistance to drugs other than isoniazid and rifampicin, although there may be potential to further develop the test to include resistance information to other anti-TB drugs. Further study should be focused on the establishment of a model for evaluating the social and economic benefits of rapid diagnosis.

### Conclusion

Overall, this study indicates that, under current conditions, Genechip is a more cost-effective method for diagnosing MDR-TB than conventional DST. However, a decrease in market price and improvements in the performance of Genechip would definitely influence further wider application of Genechip.
